# Research on the Mechanism and Control Technology of Coal Wall Sloughing in the Ultra-Large Mining Height Working Face

**DOI:** 10.3390/ijerph20010868

**Published:** 2023-01-03

**Authors:** Xuelong Li, Xinyuan Zhang, Wenlong Shen, Qingdong Zeng, Peng Chen, Qizhi Qin, Zhen Li

**Affiliations:** 1 State Key Laboratory of Mining Disaster Prevention and Control, College of Energy and Mining Engineering, Shandong University of Science and Technology, Qingdao 266590, China; 2 State and Local Joint Engineering Laboratory for Gas Drainage & Ground Control of Deep Mines, School of Energy Science and Engineering, Henan Polytechnic University, Jiaozuo 454000, China; 3 School of Mine Safety, North China Institute of Science and Technology, Langfang 101601, China; 4 Shandong Energy Group Co., Ltd., Jinan 250014, China

**Keywords:** ultra-large mining height, coal wall sloughing, numerical simulation, stability, control technology

## Abstract

One of the primary factors affecting safe and effective mining in fully mechanized mining faces with large mining heights is coal wall sloughing. This paper establishes the mechanical model of the coal wall and uses the deflection theory for the mechanics of materials to find the maximum point of the deflection of the coal wall, which is the most easily deformed and damaged during the mining process, based on the mining production conditions of the 12-2up108 working face in the Jinjitan Coal Mine. In order to simulate the characteristics of the coal wall in the large mining height working face at various mining heights, the FLAC-3D numerical method was used. The stability of the mining area was assessed in conjunction with the multi-factor fuzzy comprehensive evaluation mathematical model, and the corresponding control of the coal wall was suggested. The study demonstrates that: (1) The working surface at Jinjitan Coal Mine 112-2up108 is a typical drum-out sloughing. The coal wall is most likely to sustain damage at the point where it contacts the roof when the frictional resistance between the coal seam and the roof and floor is less than the uniform load, and at 0.578 times the mining height when the frictional resistance between the coal seam and the roof and floor is greater than the uniform load. (2) In the working face with a large mining height, mining height of the coal wall is one of the significant influencing factors. With increasing mining height, the coal wall’s height also rises nonlinearly, as does the depth of the coal wall in the working face with the large mining height. The growth is linear. The coal wall’s maximum deflection value point moves up and the slab’s height significantly increases when the mining height exceeds 7.5 m. (3) The Jinjitan Coal Mine should be supported by a pressurized and enhanced composite support bracket with a support force greater than 0.245 MPa and a support plate of 3500 mm because it belongs to a Class I stable coal wall, according to a thorough evaluation of a multi-factor fuzzy mathematical model. The working face’s mining pressure is continuously and dynamically monitored, and the stress is released in a timely manner to prevent the occurrence of dynamic disasters.

## 1. Introduction

The likelihood of coal wall sloughing increases with the increase in the mining height of coal mines, which has a significant impact on worker safety and the normal operation of shearers. The void-to-roof distance increases because of coal wall sloughing, leading to pedion roof falls and worsening roof conditions. Roof accidents occur as a result of the hydraulic support’s inability to fully support the roof, uneven forces, and deteriorating relationships with the surrounding rock. Therefore, it is crucial to use the appropriate control technology of coal wall slats for the secure production of large mining height working faces in mines through mechanical analysis, numerical simulation, and stability evaluation.

The failure mechanism of coal wall sloughing and the prevention and control methods of rib spalling have been extensively studied and applied by relevant researchers. According to Xu et al. [1], who investigated the form of coal wall instability under various constraints using the pressure bar model, weak coal seams are susceptible to shear failure and slip instability, while hard and medium coal seams are susceptible to shear failure and slip instability. Hard coal seams frequently experience collapsing failure and bending instability; Wang et al. [2,3,4] proposed three types of coal wall failure modes: compression shearing, pulling shearing, and pulling cracking; Yin et al. [5] analyzed the form and depth of the coal wall in the large mining height working face by using the stability principle of the pressure bar; Liu et al. [6,7] analyzed the rib mechanism of stepped coal cutting using numerical simulation. Suorineni et al. [8] found that the joint action of the active high stress area and the tensile stress area during the operation led to the collapse of the working face. By building a large-scale physical model in accordance with the long wall’s in-situ conditions, Lou et al. [9] studied the evolution of the long wall. In the work of Wang et al. [10], the conditions of dynamic disasters caused by the broken hard and thick rock layer (HTRS) in the fully mechanized mining face were studied by dividing the rock surrounding the roadway into three areas: the shock resistance zone (BRZ), the shock inoculation zone, and the stability zone. The splitting and caving characteristics of the rock formation were highlighted by Das et al. [11] in their study of the mechanized longwall geology, petrophysical-mechanical properties, and behavior of the coal measure roof rock during mining.

Numerous factors influence the coal wall’s large mining height sloughing. Different working face environments have different effects on the coal seam depending on the surrounding rock, its physical characteristics, and the mining support method. In the study of Wang et al. [12,13,14,15], the numerical simulation method demonstrated that the overall roof beam is more conducive to suppressing the coal wall sloughing. The Mohr-Coulomb criterion was used by Chang et al. [16] to determine the analytical expressions of the horizontal displacement of the coal wall, the radius of the rupture zone, and the plastic zone. It is suggested that the main factors of the coal wall that can be controlled are the bearing pressure concentration factor of the working face, the height of the machine mining, and the resistance of the support. In addition, Sinha et al. [17] investigated the long blocks’ effects on the block shape. The critical strain zone percentage (pcsz) and spalling zone percentage (psz) criteria were proposed by Behera et al. [18,19]. Rakesh et al. [20] determined that the thickness of the coal seam, rock mass properties, and surrounding rock stress conditions must be related to the mining of thick coal seams. Rakesh et al. [21] obtained the influence of coal pillar geological conditions and overlying rock depth on coal pillar performance [22]. Aydan et al. [23] investigated the mechanism behind the extrusion phenomenon of tunnel-encircling rocks and its associated factors.

The large mining height of the coal wall is a significant unavoidable risk in the production process, and rational and efficient management of the coal wall rib spalling is essential to ensure that the mine’s production is done safely.

According to Pang et al. [24,25,26,27], the guard plate’s force on the coal wall makes it difficult to prevent the wall from cracking, but it can prevent the destroyed body’s slip and instability after the pull. Fang et al. [28,29] considered the shear failure surface of the coal wall as a circular arc sliding surface, and analyzed the conditions of coal wall compression shear failure and put forward three principles of stability control for coal walls. Liu et al. [30] investigated the precise control technology of the entire process of intelligent mining for coal walls with a large mining height and successfully resolved the issue of intelligent mining for coal wall control [31]. By creating a mechanical model of the coal wall and applying the deflection theory from materials mechanics, the maximum point of the coal wall’s deflection is determined in accordance with the unique circumstances of the 112-2up108 working face of the Jinjitan Coal Mine. A simulation study of the situation of the coal wall with various mining heights is conducted, along with an analysis of the mechanism and regulations governing a working face with a large mining height and a recommendation for the coal wall’s corresponding control technology.

## 2. Engineering Background

**Figure 1:**
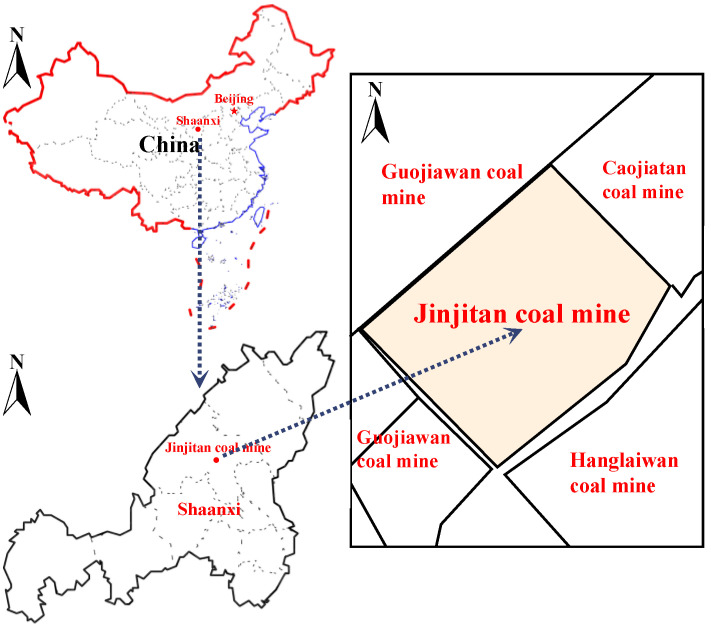
Location map of the Jinjitan coal mine.

The first working face in the southwest wing of the first panel area of the Jinjitan Coal Mine is 12-2up108. The coal seam’s thickness ranges from 5.5 to 8.4 m, with an average of 6.65 m. The coal seam’s burial depth ranges from 200 to 305 m, and its average mining depth is 246 m. The average thickness of the loose layer, which is part of the coal seam’s near-shallow buried portion, is 35 m. The mining inclination length and strike length of the 12-2up108 working face are each 300 m. The hydraulic support mining technology with an extremely high mining height is chosen for the 8.2 m fully mechanized mining process. The depth ratio is much less than 50 times, and the designed mining height is 5.8–8.2 m. The stratum is flat, the working face is elevated at +964.0 to +980.5 m above sea level, the average is +970.0 m, and the overall monoclinic structure is high in the east and low in the west, high in the south and low in the north, with no folds and magmatic activity. The relative position of the ground is mostly horizontal. It is a typical aeolian dune and beach landform covered in quaternary aeolian sand, with no surface water system passing through, as shown in Figure 1.

## 3. Mechanics Model of Coal Wall Sloughing

### 3.1. Coal Wall Sloughing Form

**Figure 2:**
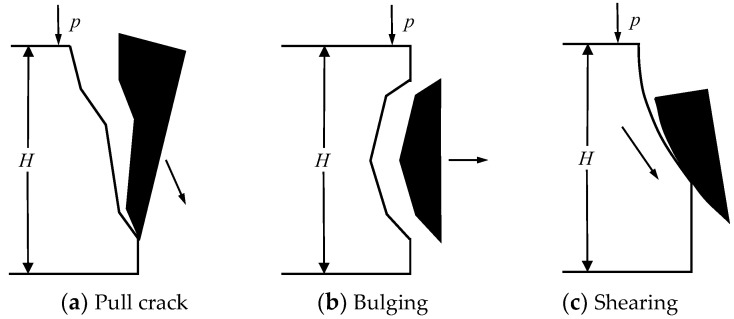
Schematic diagram of coal wall destruction under different states. P-top plate presure, H-thickness of coal seam.

For the coal wall of the working face with high coal hardness, due to the high hardness and brittleness of the coal body, the allowable deformation of the coal wall is small, and the bulging deformation of the coal wall is difficult to offset the tensile stress generated in the horizontal direction under the action of the vertical pressure; the tensile failure shown in Figure 2a occurs [32]. When the transverse tensile stress in the coal wall of the working face exceeds the tensile strength of the coal wall, the coal wall will experience bulging failure as shown in Figure 2b. For the coal wall of the working face with soft coal hardness, under the action of the self-weight of the coal body and the roof pressure, the transverse tensile stress can be released and relieved by the deformation of the coal body. Finally, because the shear stress in the coal wall is greater than the shear strength, the Shear failure as shown in Figure 2c occurs.

### 3.2. Mechanical Model of Coal Wall Stability

**Figure 3:**
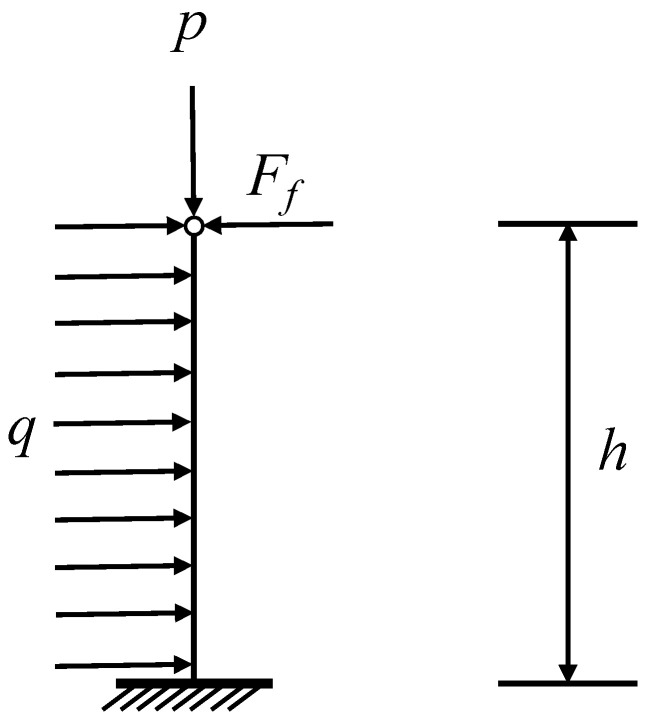
Simplified mechanical model. q-the set of horizontal loads; $F_{f}$-frictional resistance between the coal seam and the roof plate, N; P-top plate pressure, Mpa; h-mining height, m.

The coal wall is subjected to horizontal extrusion forces from the coal body in front of the work and the pressure of the top plate can be considered as an iso-sectional beam with fixed ends, simply supported at one end or free end. It is appropriately simplified to make the analysis of the deflection caused by the horizontal upward movement of the coal wall easier [33,34]: the gravity of the coal wall itself has little effect compared with the stress of the original rock, and its influence on the deflection of the coal wall is relatively small, so the gravity of the coal wall itself is not considered; the compression deformation of the coal wall in the vertical direction has less effect on its deflection, so this factor is not considered, and the simplified model is shown in Figure 3.

**Figure 4:**
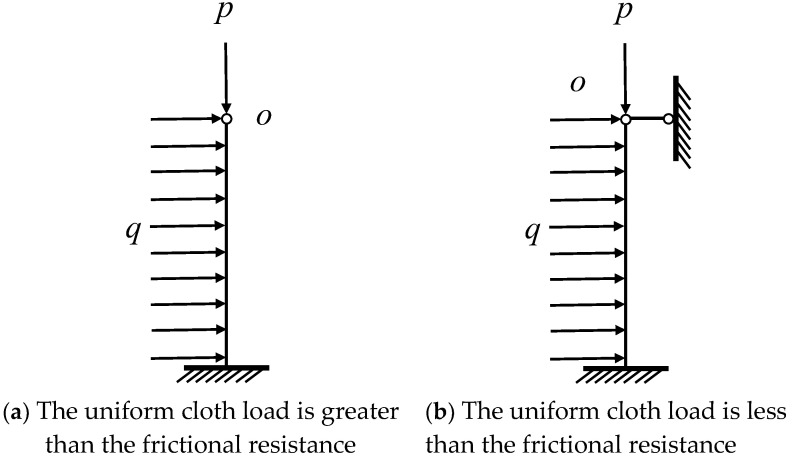
Simplified mechanical model. P-top plate pressure, q-the set of horizontal loads, O-the coordinate origin point.

Thus, there is a relationship between the frictional resistance $F_{f}$ between the coal seam and the top floor plate and the uniform load qh, assuming that $Z=qh-F_{f}$. When Z≥0, the model was simplified as shown in Figure 4a; when Z<0, the model was simplified as shown in Figure 4b.

### 3.3. Mechanical Analysis of Coal Wall Stability

**Figure 5:**
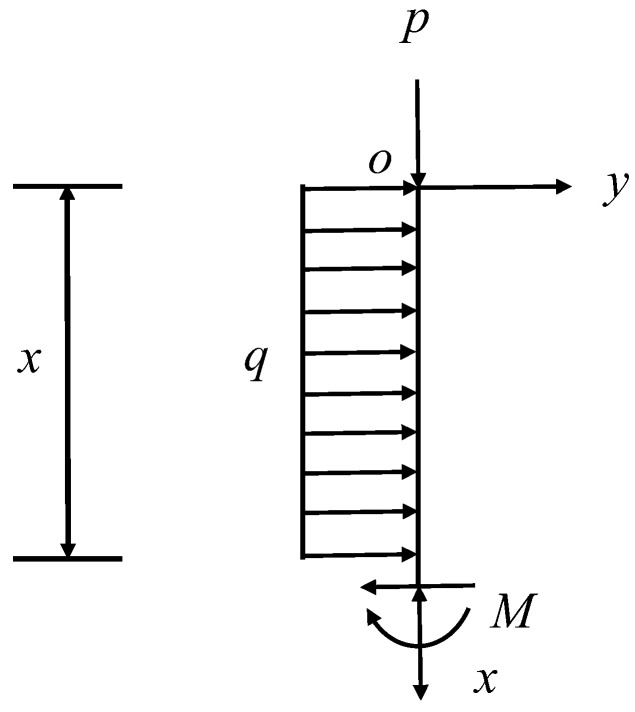
Either x-section force analysis plot. *M*- torque.

When Z≥0, the working surface coal wall can be seen as a cantilever beam with fixed ends and free ends. The beam is analyzed by force, the O point is the coordinate origin, the vertical downward is the x-axis, the horizontal right is the y-axis, the plane rectangular coordinate system is established, and any section x of the beam L is taken as the study image for force analysis, as shown in Figure 5.

This is obtained using approximate differential equations of any sectional x shaped centroid moment and deflection curve

(1) $$\omega^{\prime \prime }=\frac{qx^{2}}{2EI}$$

The deflection equation can be obtained by integrating the transformation and combining the characteristics of the cantilever beam:

(2) $$\omega =\frac{qx^{4}}{24EI}-\frac{qxh^{3}}{6EI}+\frac{qh^{4}}{8EI}$$

When x=0 deflection is maximized, the maximum deflection value point of the coal wall occurs at the contact between the coal seam and the roof plate, that is, at the mining height h, and the deflection value at this point is $\omega_{\max}=\frac{qh^{4}}{8EI}$.

**Figure 6:**
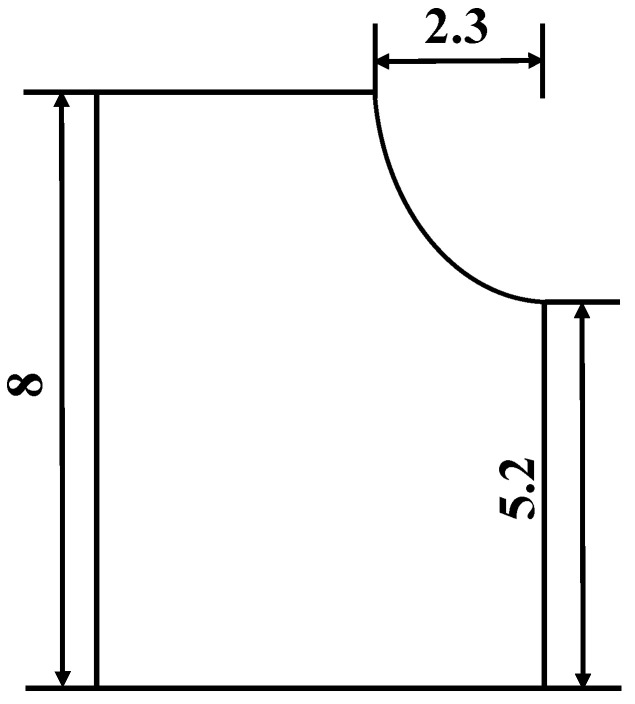
Sketch of the coal wall rib spalling.

Based on the mechanical conditions of the surrounding rock of the 8.0 m super high mining surface of the coal seam, the deflection characteristics of the coal wall are analyzed by using the pressure rod theory; the location of the coal wall prone to fragments is 5.2 m from the bottom plate, and the failure depth of the coal wall is 2.3 m, as shown in Figure 6. Under the assumption of considering friction through mechanical calculations for the 8.2 m ultra-large mining height, the support method of the bracket that was pressurized and reinforced with a composite guard mechanism is specially proposed, and the calculation is that the support force cannot be less than 0.245 MPa; the total length of the guard plate should reach 3500 mm order to significant lessen the occurrence of large coal wall sloughing on the working surface [35].

**Figure 7:**
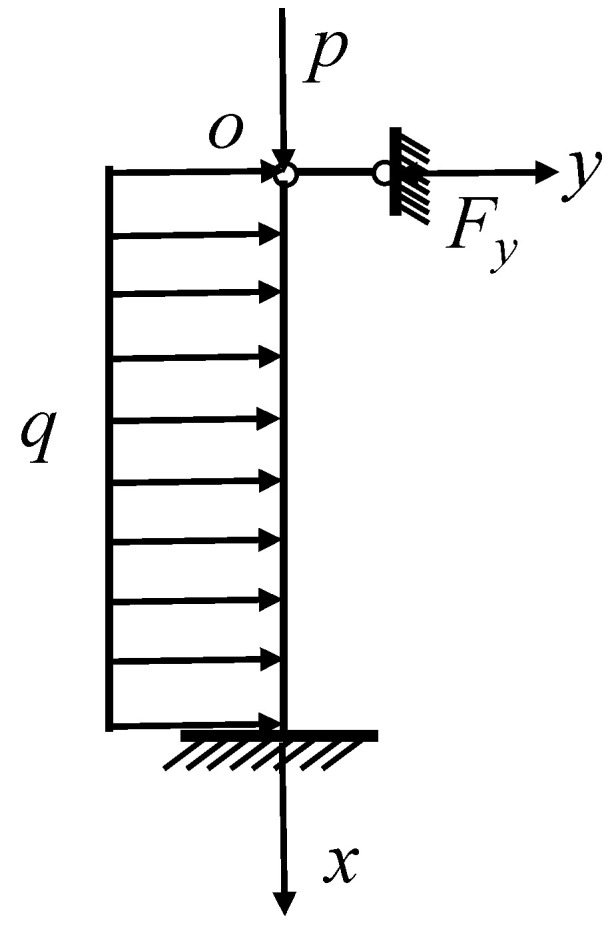
Either x-section force analysis plot.

When Z<0, that is, the equal section beam that is clamped at one end and simply supported at the other end, take the O point as the coordinate origin, vertically downward as the x-axis, and horizontally to the right as the y-axis, and establish a planar rectangular coordinate system for force analysis, as shown in Figure 7.

**Figure 8:**
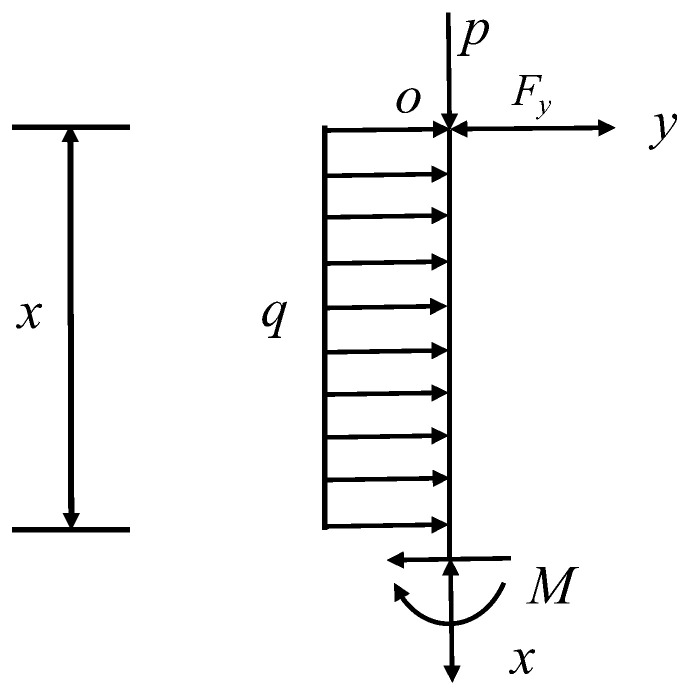
Either x-section force analysis plot.

The model is a statically indeterminate beam with an excess support force $F_{y}$ at the support O, and this excess support force is now solved [36,37,38]. Since it is a superstatically deterministic beam, the deformation compatibility condition is used here for solving, and the total deflection ω=0 is used at the O point. When calculating the deflection here, the superposition principle can be used to decompose the deflection $\omega_{op}$ of the cantilever beam that is clamped at one end and simply supported at the other end into the deflection of the cantilever beam under the action of the uniform distribution load and the pressure of the top plate; the deflection $\omega_{oF}$ of the cantilever beam under the concentrated load and the pressure of the top plate can be calculated as shown in Figure 8, and the deflection at the *O* point according to the consistency equation is zero:

(3) $$\omega_{op}+\omega_{oF}=0$$

The deflection value of the cantilever beam at the point O under a concentrated load is:

(4) $$\omega_{oF}=-\frac{F_{y}l^{3}}{3EI}$$

The deflection value of the cantilever beam at the O point under the action of the uniform distribution load q is:

(5) $$\omega_{oP}=\frac{qh^{4}}{8EI}$$

Take either x section to establish a Cartesian coordinate system for force analysis, as shown in Figure 8.

Solving the deflection equation for a superstatically deterministic cantilever beam is obtained as follows:

(6) $$\omega =\frac{qx^{4}}{24EI}-\frac{qx^{3}h}{16EI}+\frac{qh^{3}x}{48EI}$$

Through the analysis, the stagnation point needs to meet the maximum value of deflection at x⊂[0,h], so the maximum deflection value is 0.422 from the top plate, that is, 0.578 times the height of the mining height, and the deflection value of this point is:

(7) $$\omega_{\max}=\frac{13qh^{4}}{2400EI}$$

It can be concluded that the coal wall band generally occurs in the middle and upper part of the mining height, which is consistent with the actual observations at the site.

The maximum deflection point of the coal wall, that is, the position where the most likely destruction occurs, is obtained by using the deflection theory in material mechanics to analyze the mechanism of the coal wall sloughing in the large mining height comprehensive mining surface., and it is believed that there is a relationship between the friction resistance $F_{f}$ between the coal seam and the roof plate and the uniform cloth load qh, assuming that $Z=qh-F_{f}$, when Z≥0, the maximum deflection value point of the coal wall occurs at the contact between the coal seam and the top plate, and the deflection value at this point is $\omega_{\max}=\frac{qh^{4}}{8EI}$, and the coal wall is most likely to be destroyed; when Z<0, the maximum deflection value of the coal wall occurs at 0.578 times the mining height, the deflection value at this point is $\omega_{\max}=\frac{13qh^{4}}{2400EI}$, and the coal wall is most prone to destruction.

## 4. Numerical Simulation Study of Large Mining Height Coal Wall Sloughing

As one of the main coal mining methods for safe and efficient mining in coal mines, large-scale mining and high-variety mining are high-scale mining. When the working surface is not excavated, the coal body is in the state of original rock stress, and the coal wall shows a certain continuity. The original rock stress balance of the coal body is broken with the recovery of the working surface, the horizontal stress of the coal wall is rapidly reduced, the support pressure effect causes the vertical stress of the coal wall to rapidly increase, the coal wall of the working surface is destroyed, and new joints and fractures are generated [39,40,41].

### 4.1. Model Building

In the numerical model, the mechanical parameters taken by each layer of rock mass are simulated, and the mechanical parameters of the drill hole are based on the comprehensive column chart of the borehole, the contour diagram of the buried depth of the coal seam [42] and the rock mechanical parameters, as shown in Table 1.

**Figure 9:**
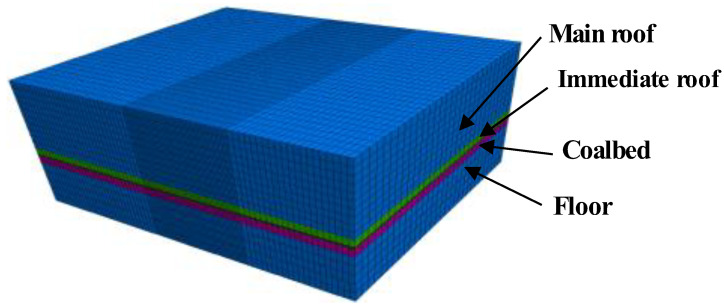
A diagram of a 3D numerical model.

The bottom boundary of the model is subject to horizontal and vertical constraints, and the mesh is calculated according to the model’s geometric dimensions, considering the restriction of the total number of model elements and the concentration of the problem area under study. The numerical simulation model [43,44] is shown in Figure 9, and the mode of the simulation can be seen.

### 4.2. Simulation Results

The advanced support pressure of the original rock stress will appear on the coal body with the forward movement of the working surface of large-scale mining and high comprehensive mining [45,46,47], and the simulation results show that under the action of this high stress, the elastic region, plastic zone, and proto-rock stress region appear at the edge of the coal body [48], and the stress transfer to the interior of the coal.

**Figure 10:**
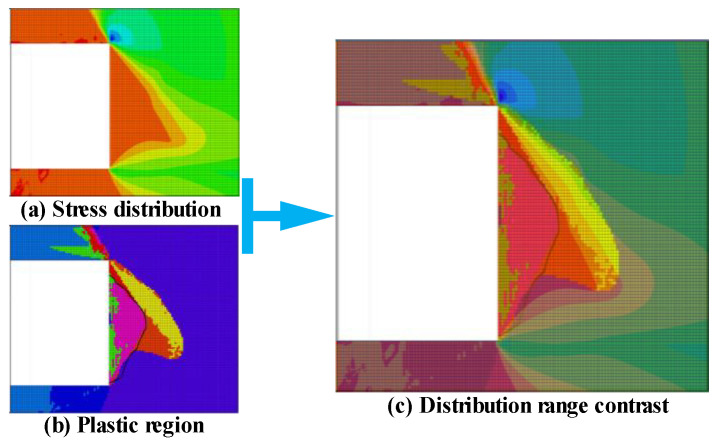
Comparison of the ranges of plastic and stress zones in the coal wall.

The coal body is destroyed when the concentrated stress is greater than its tensile strength, and it is essentially in a stable state when the concentrated stress is equal to or less than its bearing capacity [49,50]. By using the mining height of 7.5 m as an example (Figure 10), the range of the plastic area and the stress area of the coal wall is compared and analyzed, and the maximum main stress area near the coal wall of the working surface is divided into the tensile stress area and the compressive stress area as well as the plasticity near the coal wall of the working surface is divided into the shear plastic area and the tensile plastic area. The maximum tensile stress area covers all the tensile plastic areas and part of the shear plastic area, and the tensile plastic area produces a bulging form of the tablet under the action of tensile stress.

**Figure 11:**
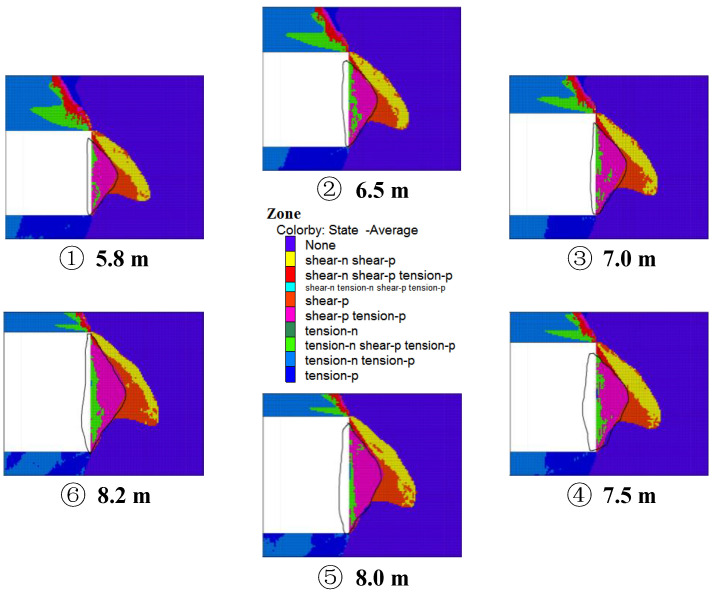
Distribution of rock mass plasticity zones in different mining height coal seams.

Figure 11 shows the distribution map of the plastic area of the coal wall mining in different mining high coal seams, and during the propulsion of the large mining height working surface, the working surface coal wall is damaged within a certain range under the action of support pressure, along the direction of propulsion. The increase in the mining height increases the sinking space of the top plate of the goaf area, so that the plastic area of the working surface coal body and the surrounding rock is more developed, and under the action of support pressure, the reduction of the coal body’s own strength leads to the overflow of the coal wall of the working surface, resulting in the coal wall sloughing.

Figure 11 shows the distribution map of the plastic coal wall mining area in various high coal seams. When the large mining height working surface is propelled, the coal wall mining area is damaged within a specific range as a result of support pressure acting in the direction of propulsion. The plastic area between the working surface coal body and the surrounding rock is further developed because of the increased mining height, which also increases the sinking space of the top plate of the goaf area. When support pressure is applied, the working surface coal body’s own strength is reduced, which causes the coal wall to overflow and slough.

**Figure 12:**
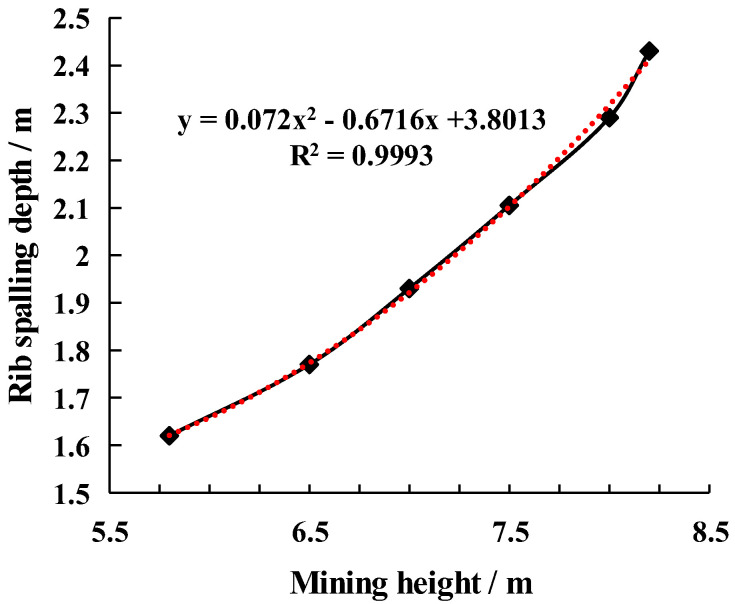
Fitting curve graph of the relationship between the coal seam mining height and coal wall band depth.

The coal wall band situation is determined by calculating the maximum tensile stress of the coal wall during coal seam mining and the corresponding range of tensile plasticity zone of coal seams (see Figure 12). (1) The height of the mine is 5.8 m; the sheet depth is 1.61 m; and the height is 5.00 m; (2) The height of the mine is 6.5 m; the sheet depth is 1.86 m; and the height is 5.72 m; (3) The height of the mine is 7.5 m; the sheet depth is 2.18 m; and the height is 6.53 m; (5) The height of the mine is 8.0 m; the depth of the film is 2.27 m; and the height is 7.05 m; (6) the height is 8.2 m; the depth of the film is 2.39 m; and the height is 7.32 m.

The depth of the coal wall sloughing of the large mining height working surface increases with the mining height rather than linearly, as can be seen from the relationship curve between the mining height of the coal seam and the coal wall sloughing; the fitting curve is “$y=0.072x^{2}-0.6716x+3.0813$”, which indicates that the mining height is one of the significant influencing factors of the coal wall band of the large mining height working surface.

**Figure 13:**
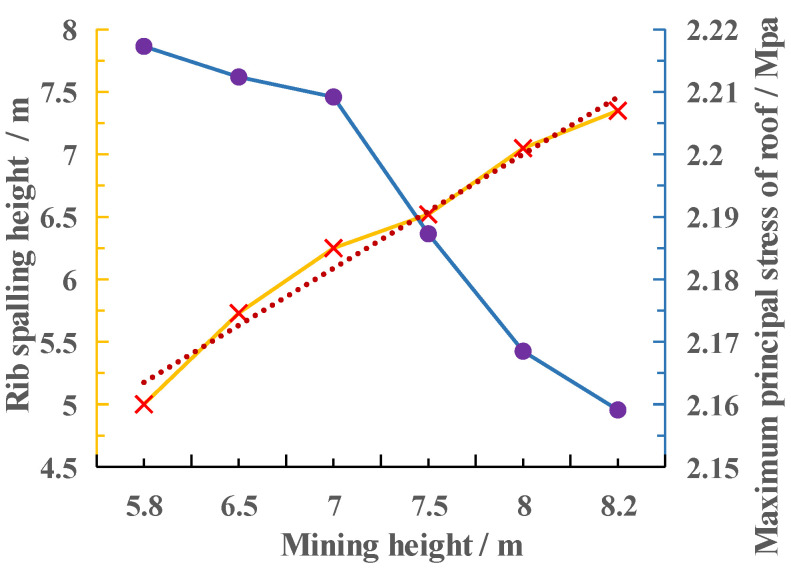
Diagram of the maximum principal stress relationship between coal seam mining height and coal wall plate height, and coal seam and roof plate. Blue line is the maximum principal stress, light red is the rib spalling height.

Additionally, the results of the numerical calculations demonstrate that the height of the coal wall sloughing, the maximum main stress between the coal seam and the top plate, and the mining height of the coal seam have a clear relationship with one another (see Figure 13). The line chart demonstrates that when the mining height is greater than 7.5 m, the height of the sloughing increases with the mining height. The maximum main stress between the coal seam and the top plate decreases with the mining height, and when the mining height is greater than 7.5 m, the maximum principal stress between the coal seam and the top plate decreases rapidly with the increase in the mining height. This is due to the fact that the maximum principal stress and frictional resistance between the coal seam and the roof plate are positively correlated, that the change in mining height is minimal compared to the overburden thickness, that the uniform load of the coal seam remains unchanged, that the frictional resistance decreases with increasing mining height, that the frictional resistance decreases by an increased amplitude at 7.5 m, and that the maximum deflection value point of the coal seam is located at this height.

## 5. Coal Wall Stability Evaluation and Chip Control Technology

### 5.1. Multi-Factor Fuzzy Comprehensive Assessment Model

The mechanical characteristics of the coal seam itself and the conditions of the overlying rock layer are related to the stability of the coal wall, and the conditions of the overlying rock layer determine the supporting pressure characteristics, and the model determines the correlation between the five types of coal seam indicators and the stability of the coal wall [51]. (1) The firmness of coal: the hardness index of coal measured using the mashing method has good convergence, which can basically reflect the anti-external force characteristics of the coal matrix; the hardness index also considers the influence of micro-fissures and coal brittleness, and is easy to determine. (2) The compressive strength of coal: due to its large dispersion, it can be used as an auxiliary index for the mechanical characteristics of coal seams. (3) The degree of macroscopic fracture development of coal: as shown in Table 2, the relationship between the fracture and working surface can be divided into six categories. Sort the tables as 1 and 2 into one category, and give a score of 80 to 100 in advance; sort 3 is the second category, with a score of 70 to 80; sort 4 is the third category, with a score of 60 to 70; and sort 5 and 6 are the fourth category, with a score of 50 to 60. (4) The thickness of hard layering in coal. (5) Hard layered position: the above five factors are quantified as shown in Table 2.

The set of evaluation factors for the classification of coal wall stability [52] is:

(8) $$U=\left\{U_{1}\right.,U_{2},U_{3},U_{4},\left.U_{5}\right\}=\left\{f\right.,R_{c},\lambda ,X,\left.D\right\}$$

The set of categories for coal wall stability is:

(9) $$\begin{array}{l} V=\{V_{1},V_{2},V_{3},V_{4}\}=\{I,II,III,IV\}\\ =\{\mathrm{stable},\;\mathrm{relatively}\;\mathrm{stable},\;\mathrm{moderate}\;\mathrm{stable},\;\mathrm{unstable}\} \end{array}$$

The one-factor evaluation ${\bar{R}}_{i}=(r_{i1},r_{i2},r_{i3},r_{i4})$ of the ith factor is regarded as a fuzzy subset on the domain V, and the evaluation factors affecting the stability of the coal wall should be weighed in general. The weight distribution between the factors can be established, and when the total fuzzy evaluation matrix $\bar{R}$ of the weight vector is known, according to the fuzzy linear weighted transformation method, matrix $\bar{B}$ can be evaluated and normalized:

(10) $$\bar{B^{\prime }}=(\frac{b_{1}}{b}+\frac{b_{2}}{b}+\frac{b_{3}}{b}+\frac{b_{4}}{b})=(b_{1}^{\prime },b_{2}^{\prime },b_{3}^{\prime },b_{4}^{\prime })$$

where $b=\sum b_{j}$, the stability of the coal wall can be evaluated by judging the size of $b_{j}^{\prime }(j=1,4)$ in matrix $\bar{B^{\prime }}$.

The comments are quantified on a 100-point scale, and the scoring criteria are: *C_I_* = 100, *C_II_* = 80, *C_III_* = 60, *C_IV_* = 40.

The overall evaluation score is:

(11) $$\bar{S}=\bar{B^{\prime }}\cdot {\bar{C}}^{T}=(b_{1}^{\prime },b_{2}^{\prime },b_{3}^{\prime },b_{4}^{\prime }){\left(C_{I},C_{II},C_{III},C_{IV}\right)}^{T}$$

Finally, the coal wall stability classification is carried out according to the score size. The classification criteria are:

Class I: $\bar{S}>90$; Class II: $\bar{S}=80\sim 90$; Class III: $\bar{S}=70\sim 80$; Class IV: $\bar{S}>60\sim 70$

### 5.2. Rating Factor Set Membership Function

The basis of the comprehensive evaluation of multi-factor fuzziness is the one-factor fuzzy assessment, so the membership function of each assessment factor for the $\bar{S}=\bar{B^{\prime }}\cdot {\bar{C}}^{T}=(b_{1}^{\prime },b_{2}^{\prime },b_{3}^{\prime },b_{4}^{\prime }){\left(C_{I},C_{II},C_{III},C_{IV}\right)}^{T}$ qualitative category of coal wall stability is established.

For this classification, in the array $\mu_{{\bar{R}}_{i}}(U_{i},V_{1})$, $\mu_{{\bar{R}}_{i}}(U_{i},V_{2})$, … $\mu_{{\bar{R}}_{i}}(U_{i},V_{4})$, if the $\mu_{{\bar{R}}_{i}}(U_{i},V_{j})$ is the maximum value, it can be only for the first $U_{i}$ factor, and it should be the $V_{j}$ level. In general, in addition to satisfying $\mu_{{\bar{R}}_{i}}(U_{i},V_{j})$ = 1, the $\mu_{{\bar{R}}_{i}}(U_{i},V_{j})$ strain is small when the $U_{i}$ is far from where the $U_{i}$ is located. Therefore, the membership function of the factors that influence coal wall stability on the coal wall stability category can be taken as a normally distributed function, that is

(12) $$\mu (U)=\exp^{\left\{-{\left[(U-m)/t\right]}^{2}\right\}}$$

where m and t are constants.

When the boundary value of each level of physical quantity range is between the two levels, the membership of both levels is the same, so that it is approximately equal to 0.5, which is:

(13) $$\exp^{\left\{-{\left[(U_{up}-U_{down})/2t\right]}^{2}\right\}}=0.5$$

where $U_{up},U_{down}$ are the upper and lower boundary values of the physical quantities of that level, respectively.

The parameter values of t in the membership function can be derived as shown in Table 3.

When we know the $U_{i}$ of the value of the factor set of each coal seam, we can find each membership $\mu_{\bar{R}}(U)$ according to the m and t values in Equation (12) and Table 3, which are the elements $r_{ij}$ in the fuzzy relationship matrix $\bar{R}$.

### 5.3. Evaluate the Factor Weight Vector $\bar{A}$

The weights of each influencing factor in the stability classification are:

(14) $$\bar{A}=\frac{A_{1}}{f}+\frac{A_{2}}{D}+\frac{A_{3}}{\lambda }+\frac{A_{4}}{X}+\frac{A_{5}}{R_{c}}=\left(0.4,0.3,0.2,0.15,0.05\right)$$

The purpose of stability classification is to evaluate the comprehensive conditions of coal seam thickness suitable for a large mining height, as one of the main evaluation indicators for the use of large mining height technology. According to the above mathematical model and classification method, the comprehensive score of the Jinjitan Coal Mine is 95.9, indicating a class I stable coal wall. The actual production proves that the mine is suitable for the use of a large mining height, and the mining height can be large.

### 5.4. Coal Wall Sloughing Control Technology

According to the above analysis and discussion, the main reason for the coal wall band is that it is internally destroyed under the pressure of the top plate and the stress release occurs at the maximum deflection value point of the coal wall under the action of tension stress. Hence, the following suggestions are proposed for the control of the coal wall band on the 12-2up108 working surface of the Jinjitan Coal Mine:

(1) Reasonable control of mining height

The study shows that the coal seam mining height is closely related to the depth and height of the coal wall sloughing. As the working surface is increased, the probability of the coal wall sloughing increases and more serious sloughing occurs, after which the front beam and the guard plate of the bracket cannot play their role very well. Therefore, on the working surface with poor stability of the coal wall, an appropriate method of reducing the mining height should be adopted to reduce the phenomenon of coal wall fragments.

(2) Timely and effective support

The choice of a timely and reasonable support method and the use of a high initial support hydraulic bracket are conducive to sharing the pressure of the top plate above the working surface, and reduce the effect of reducing the pressure on the coal wall. The uniform distribution load between the coal wall and the top plate is reduced and the frictional resistance increases. Through mechanical calculation, the pressurized reinforced composite guard bracket should be used for support, and the protective force is greater than 0.245 MPa; the total length of the guard plate should reach 3500 mm, thereby reducing the occurrence of the coal wall banding phenomenon.

(3) Accelerate the speed of the working surface

The coal body itself has creep characteristics. When the large mining height working surface is advanced, the working surface coal wall produces damage within a certain range of the propulsion direction. The appropriate acceleration of the propulsion speed of the working surface can reduce the scope of coal wall destruction, improve the stability during the operation of the working surface, and reduce the probability and depth of the coal wall.

(4) All-round continuous dynamic monitoring of working surface ore pressure

Using online pressure monitoring equipment, the working surface mining pressure is continuously monitored in an all-round way, combined with theoretical calculations, all of which are used for predicting and forecasting the working surface top plate pressure time, and taking effective measures to reduce the degree of the piece during the working surface pressure.

## 6. Conclusions

Coal wall destruction and stress release are two processes that are involved in coal wall fragment disasters of large mining height working surfaces, and the two have a certain relationship. By examining the connection between various mining heights and coal wall deformation and destruction, the phenomenon of coal wall fragments on the working surface of large mining heights is addressed, and the following conclusions are drawn:

(1) The coal rock sloughing type of the 12-2up108 working surface of the Jinjitan Coal Mine is a typical bulging form of the sloughing, and the maximum deflection value point in the mechanical model of the coal wall is determined using the deflection theory in material mechanics. The results demonstrate that the coal wall’s maximum deflection value point occurs when the frictional resistance between the coal seam and the top floor plate is less than the uniform distribution load. The maximum deflection appears at the contact between the coal seam and the top plate. The coal wall is most easily destroyed, and the deflection value at this point is $\omega_{\max}=\frac{qh^{4}}{8EI}$. When the friction resistance between the coal seam and the top bottom plate is greater than the uniform cloth load, the maximum deflection value point of the coal wall occurs at 0.578 times the mining height, and the deflection value at this point is $\omega_{\max}=\frac{13qh^{4}}{2400EI}$. In this condition, the coal wall is most prone to destruction.

(2) The mining height is one of the significant influencing factors of the sloughing of large mining height working surfaces of coal walls. The depth of the sloughing of large mining height working surfaces of coal walls increases with the mining height rather than linearly; the fitting curve is “$y=0.072x^{2}-0.6716x+3.0813$”. As mining height increases, the height of the coal wall sloughing increases linearly, except when the mining height is greater than 7.5 m. The increase in the height of the sheet becomes larger, which is mainly because of the friction resistance between the coal seam and the top plate, and this value decreases to a greater extent when the mining height is greater than 7.5 m. Then, the difference between the friction resistance and the average cloth load becomes smaller, and the maximum deflection value of the coal wall moves up.

(3) Coal wall stability evaluation, one of the key evaluation indicators for the use of large mining and high comprehensive mining technology, when combined with reasonable prevention and control technology can effectively ensure the safe production of the working surface. The Jinjitan Coal Mine’s comprehensive score, 95.9, places it in the class I stable coal wall category following the evaluation of a multi-factor fuzzy mathematical model. Thus, the mine’s large mining height working surface should be supported by a pressurized and reinforced composite guard bracket with a protective force greater than 0.245 MPa and a 3500 mm guard plate. At the same time, the working surface ore pressure should also be continuously and dynamically tracked in all directions.

## Figures and Tables

**Table 1:** JB22 brief list of petrophysical and mechanical properties of drilled coal seams and their overburdens.

Number	Rock Name	Thickness	Depth	Compressive Strength (MPa)	Tensile Strength (MPa)	Modulus of Elasticity (GPa)	Poisson’s Ratio	Adhesion (MPa)	Bulk Weight
M 12	Coarse-grained sandstone	3.28	181.45	64.81	6.50	12.35	0.23	3.97	2.50
M 11	Siltstone	2.60	184.73	59.65	5.67	14.62	0.22	4.27	2.35
M 10	Mudstone	6.17	187.33	46.12	3.55	8.67	0.21	2.04	2.25
M 9	Siltstone	2.00	193.50	69.54	7.02	14.62	0.22	4.83	2.50
M 8	Fine-grained sandstone	3.00	195.50	69.65	6.97	14.62	0.22	4.27	2.35
M 7	Mudstone	5.03	198.50	46.12	3.55	8.67	0.21	2.04	2.25
M 6	Fine-grained sandstone	3.20	203.53	65.55	6.58	13.64	0.23	4.66	2.35
M 5	Siltstone	12.10	206.73	69.65	6.97	14.62	0.22	4.27	2.50
M 4	Fine-grained sandstone	9.22	215.95	65.55	6.58	13.64	0.23	4.66	2.35
M 3	Coarse-grained sandstone	15.23	231.18	64.81	6.50	12.35	0.23	3.97	2.50
M 2	Fine-grained sandstone	5.92	237.10	59.54	5.62	14.62	0.22	4.83	2.35
M 1	Siltstone	2.21	239.31	59.65	5.67	14.62	0.22	4.27	2.35
M 0	2-2 coals	7.89	247.20	12.10	1.10	1.86	0.28	0.75	1.5
F 1	Siltstone	1.23	248.43	59.65	5.67	14.62	0.22	4.27	2.35

**Table 2:** Fuzzy classification factor index of stability of large mining height coal walls.

Classify	Indicators Solidity Coefficientf		$\mathrm{Strength}R_{c}/\mathrm{MPa}$		Hard Layer Thickness/Whole Layer Thickness/λ		Hard Layer Position (Distance from Top Plate/m)X		Macroscopic Fracture Development DegreeD	
	Range	Average	Range	Average	Range	Average	Range	Average	Range	Average
I	4~12	8	25~35	30	0.8~1	0.9	0~2	1	80~100	90
II	3~4	3.5	20~25	22.5	0.5~0.8	0.65	2~3	2.5	70~80	75
III	1~3	2	10~20	15	0.3~0.5	0.4	3~4	3.5	60~70	65
IV	<1	0.5	<4~10	7	0.1~0.3	0.2	≥4~6	5	50~60	55

**Table 3:** Related parameters in membership functions.

**Category**	f		$R_{c}$		λ		X		D	
	m	t	m	t	m	t	m	t	m	t
I	8	4.82	30	6.02	0.9	0.12	1	1.	90	12.05
II	3.5	0.60	22.5	3.02	0.65	0.18	2.5	20.6	75	6.02
III	2	1.2	15	6.02	0.4	0.12	3.5	0.6	65	6.02
IV	0.5	0.60	7	3.61	0.2	0.12	5	1.2	55	6.02

## Data Availability

All data of this study have been included in the text.
